# Superior Mesenteric Artery Syndrome: The Dark Side of Weight Loss

**DOI:** 10.7759/cureus.1859

**Published:** 2017-11-18

**Authors:** Sakshi Sahni, Malan Shiralkar, Safra Mohamed, Robert Carroll, Barbara Jung, Ron Gaba, Cemal Yazici

**Affiliations:** 1 Department of Medicine, University of Illinois at Chicago College of Medicine; 2 Department of Radiology, University of Illinois at Chicago College of Medicine

**Keywords:** superior mesenteric artery syndrome, small bowel obstruction, rapid weight loss, duodenojejunostomy

## Abstract

Superior mesenteric artery (SMA) syndrome is a rare cause of small bowel obstruction (SBO) resulting from compression of the duodenum by the SMA. Patients at risk of developing SMA syndrome include those who have experienced rapid weight loss from chronic illnesses, malignancy, bariatric surgery, eating disorders, burns, trauma, or substance abuse. We present the case of a 54-year-old cachectic female patient who presented with sudden onset nausea, vomiting, and severe epigastric pain. Imaging studies revealed distention of the stomach and proximal portion of the duodenum with abrupt narrowing of the third part of the duodenum consistent with SMA syndrome. A laparoscopy confirmed the diagnosis and duodenojejunostomy resulted in resolution of the symptoms.

## Introduction

Superior mesenteric artery (SMA) syndrome is a rare cause of small bowel obstruction (SBO) first described by Rokitansky in an anatomy textbook in 1842 [1]. Upon extensive literature review, we found a total of 423 cases of SMA syndrome reported in the English language. SMA syndrome is caused when the duodenum is compressed between the aorta and the SMA. The SMA emerges from the anterior aspect of the aorta at the first lumbar vertebral level, where it is encased in the adipose and lymphatic tissue. When the angle and distance between the aorta and SMA is reduced such as in the case of weight loss, it can cause compression of the duodenum. Patients can experience a wide spectrum of symptoms ranging from postprandial nausea and pain to complete bowel obstruction. We present a case of SBO caused by SMA syndrome in a cachectic woman, eventually requiring surgical intervention.

## Case presentation

**Figure 1:**
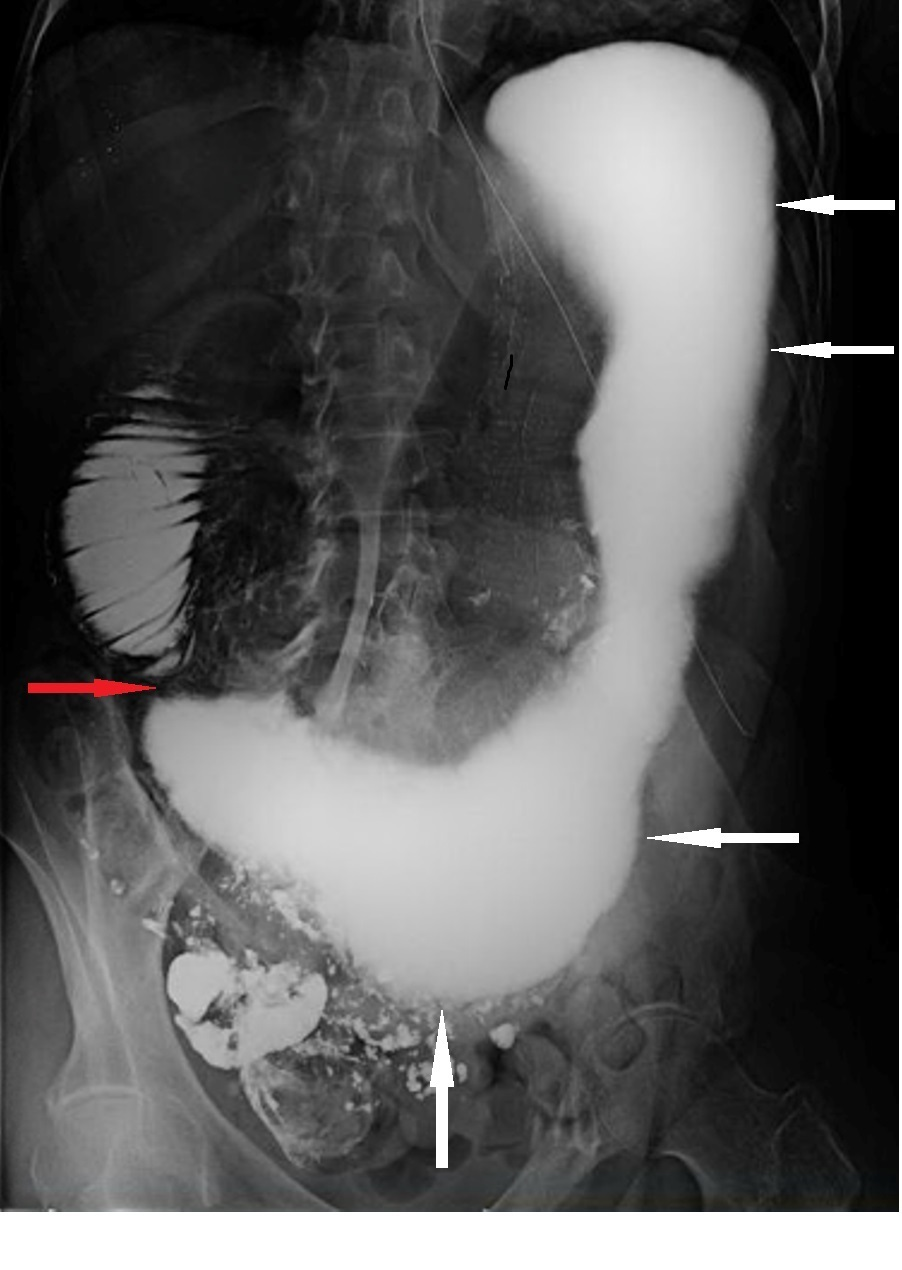
Barium swallow consistent with possible small bowel obstruction (SBO). The figure illustrates a barium swallow study showing distention of stomach and proximal duodenum (white arrows) with abrupt narrowing of the third part of the duodenum (red arrow). Radiographic findings with slow passage of contrast led to high suspicion of SBO.

**Figure 2:**
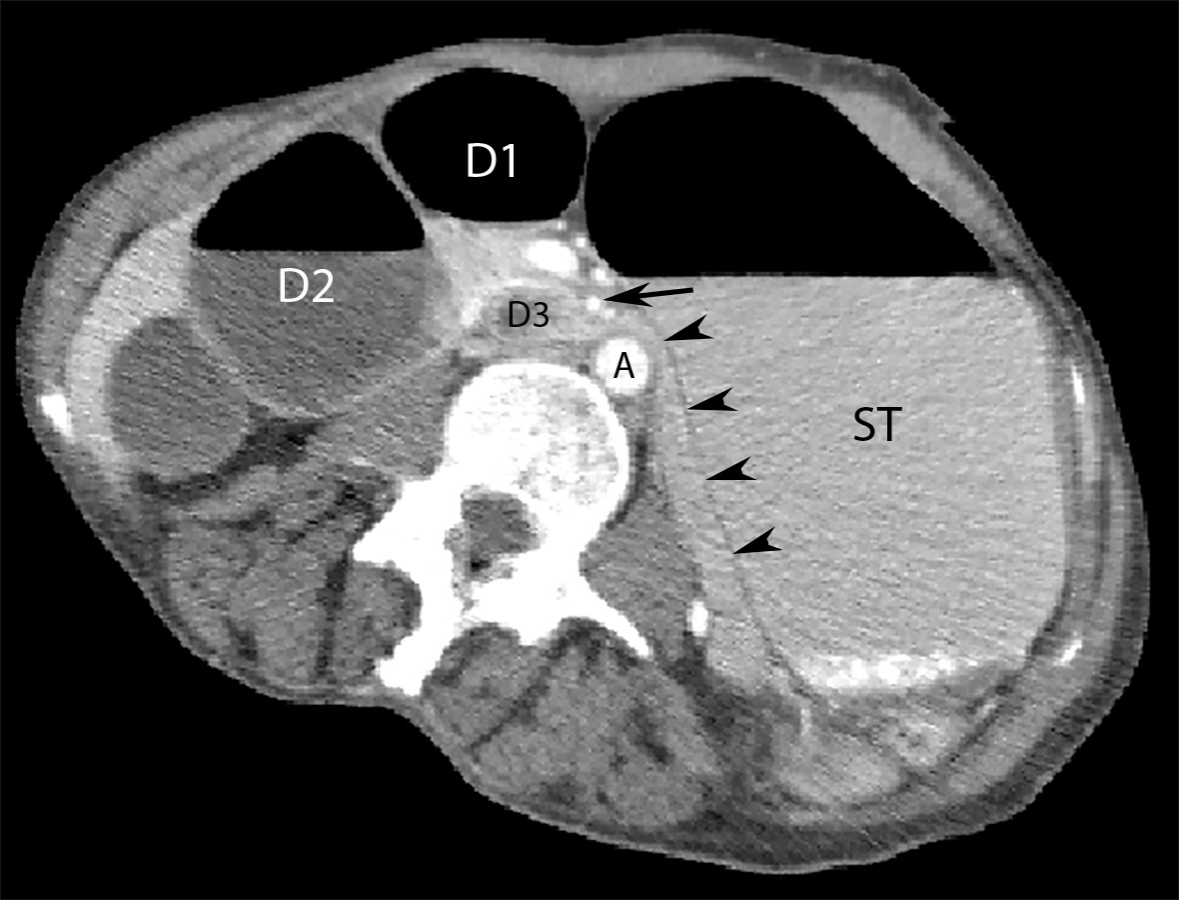
Computed tomography (CT) scan (axial view) of abdomen raising suspicion of superior mesenteric artery (SMA) syndrome. The figure depicts a computed tomography (CT) scan of abdomen showing dilatation of stomach (ST) and the first (D1) and second (D2) part of duodenum with abrupt collapse of the third part of duodenum (D3) at the point where it crossed posterior to SMA (arrow). Arrowheads also represent the collapsed duodenum.

**Figure 3:**
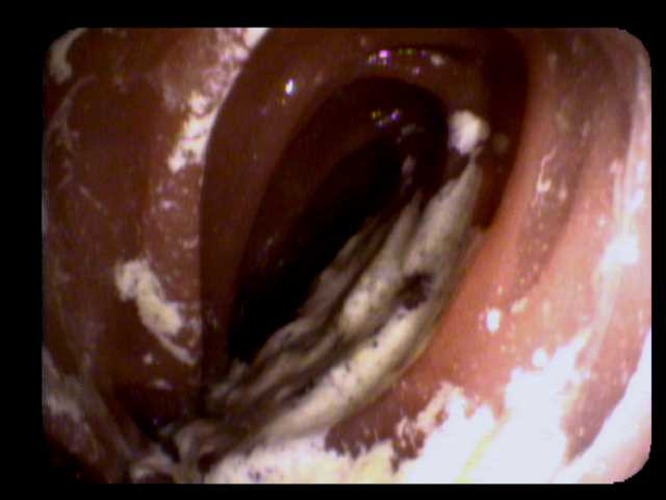
Image of the proximal portion of duodenum during esophagogastroduodenoscopy (EGD). The figure shows an image taken during EGD further demonstrating dilatation of the proximal portion of the duodenum.

A 54-year-old female with a history of untreated multiple sclerosis and substance abuse presented with sudden onset nausea, vomiting, and abdominal pain. On examination, she was noted to be cachectic with a body mass index of 15 kg/m^2^. Abdominal examination revealed high-pitched bowel sounds and a distended, diffusely tender abdomen. Her serum potassium was 3.2 mmol/L, and she had total serum protein of 4.6 g/dL and albumin of 2.4 g/dL indicating poor nutritional status. She was found to have SBO as barium swallow (Figure 1) and computed tomography (CT) scan (Figure 2) of abdomen and pelvis revealed a distended stomach and first half of the duodenum with abrupt narrowing of the third part. She underwent nasogastric tube decompression with drainage of approximately seven liters of bilious fluid. The patient was aggressively treated with intravenous fluids along with appropriate repletion of the electrolytes. She subsequently underwent esophagogastroduodenoscopy (EGD) (Figure 3), which showed a dilated stomach and proximal portion of duodenum concerning for SMA syndrome. Nasojejunal tube was placed during the endoscopy for enteral nutrition. Total parenteral nutrition (TPN) was started two weeks after the admission as the patient was unable to tolerate enteric feeds and had no improvement in her symptoms. She subsequently required surgical intervention due to the lack of clinical improvement despite the medical management and nutritional support. During the diagnostic laparoscopy, she was found to have a massively dilated stomach and duodenum up to the third portion consistent with SMA syndrome. Several clinical clues supported the diagnosis of SBO due to SMA syndrome. These included the presence of multiple risk factors such as significant weight loss and cachexia in the setting of poor oral intake and underlying neurological disorder. In addition to the presence of obstructive symptoms seen in SMA, laboratory findings indicated poor nutritional status and imaging studies (i.e., barium study and CT scan) demonstrated SMA as the etiology. Subsequently, a duodenojejunostomy was performed. Following this procedure, the patient reported improvement in her symptoms, and was able to tolerate oral feeds without significant nausea, vomiting, or postprandial abdominal pain.

## Discussion

SMA syndrome is a rare cause of obstruction of the third portion of duodenum resulting from the compression by SMA. Predisposing factors include rapid weight loss from chronic illnesses, malignancy, bariatric surgery, eating disorders, burns, and substance abuse [2-4]. It has also been shown to be associated with prolonged supine positioning, high insertion of the ligament of Treitz, trauma, and use of spinal orthosis and body casts [3]. The usual angle between the SMA and aorta ranges from 28 to 65 degrees, leaving approximately 10 to 34 mm of distance in which the duodenum passes. Patients with acute weight loss may experience acute loss of the mesenteric fat pad, causing the angle and the distance between the two vessels to decrease, resulting in compression of the duodenum. Our patient’s history of substance abuse, untreated multiple sclerosis, and cachexia from poor nutrition with an approximate unintentional weight loss of 37 lbs over one year placed her at risk of developing SMA syndrome.

Risk factors for mesenteric ischemia include dislodged thrombus due to underlying cardiac disease, significant smoking history, atherosclerotic disease, hypercoagulable or pro-thrombotic states, hypoperfusion, and splanchnic vasoconstriction. Our patient did not have these risk factors. Chronic mesenteric ischemia classically presents with postprandial abdominal pain more insidious in onset, causing the patient to develop food fear and subsequently lose weight; they usually do not have obstructive symptoms [5]. On the other hand, patients with acute mesenteric ischemia present with sudden onset severe abdominal pain out of proportion to the physical examination findings. However, the signs and symptoms can significantly vary depending on the timing of the presentation and the underlying etiology. In contrast, SMA syndrome is most commonly associated with significant weight loss leading to the loss of mesenteric fat pad as a consequence of medical disorders, psychological disorders, anatomic abnormalities, or surgery (e.g., bariatric surgery or corrective surgery for scoliosis). Symptoms are consistent with proximal small bowel obstruction; if more advanced, patients develop severe nausea, bilious emesis, and further weight loss [6].

Our patient not only had the underlying neurological disorder and weight loss but also presented with the above-mentioned symptoms. In SMA syndrome, oral contrast studies including upper gastrointestinal series and CT scan show significant dilation of stomach and duodenum associated with marked and abrupt delay in the passage of the contrast from the third part of the duodenum into the distal bowel [7]. These features were present both on barium swallow as well as CT scan in our patient. On the other hand, CT scan in patients with mesenteric ischemia may show focal or segmental bowel wall thickening, intestinal pneumatosis, mesenteric stranding, or porto-mesenteric thrombosis. Bowel dilatation may also be seen in mesenteric ischemia, but involvement of the stomach or abrupt cut-off are not characteristic features [5].

As mentioned in our patient, radiographic findings include a widened duodenum proximal to a sharp obstruction at the point where the SMA causes compression of the duodenum. Placing the patient in knee-chest position during a contrast study may allow the passage of contrast to the point where there was previously no passage [8]. Ultrasound can also be used to measure the angle between the aorta and SMA, and may show positional variation in the angle.

Other diseases that mimic SMA syndrome include duodenal dilation and decreased motility from scleroderma and collagen vascular diseases, as well as malrotation with duodenal obstruction by the congenital bands [8].

Initial treatment includes correction of the underlying causes by providing nutritional support, encouraging weight gain, and postural therapy [3]. Nutrition is an important component of conservative management in patients with SMA syndrome. Nutritional support is specially required in the initial stages until the patients are able and willing to increase their oral intake. It is usually provided by placing a nasojejunal feeding tube distal to the site of obstruction [9]. Total parenteral nutrition may be used in patients who are not able to tolerate enteral feeds or when aiming for higher calorie supplementation. Surgery is indicated when conservative measures fail. Nutritional status should be re-evaluated prior to the surgery in order to ensure a successful outcome.

There are three types of surgery for the treatment of SMA syndrome. Strong’s procedure is the least invasive surgery as it does not require bowel anastomosis. The procedure involves dividing the ligament of Treitz to mobilize the duodenum to be positioned to the right of the SMA, avoiding duodenal compression [4]. However, Strong’s procedure has fallen out of favor as it has a high failure rate. The high failure rate is presumably due to short branches of the inferior pancreaticoduodenal artery not permitting the duodenum to fall inferiorly [10]. The most commonly performed as well as the most successful surgery is duodenojejunostomy. Gastrojejunostomy can also be performed if gastric distention persists.

## Conclusions

The diagnosis of SMA syndrome is often missed as this is a fairly rare entity. There remains some controversy about the diagnosis of SMA syndrome as symptoms of the disorder do not necessarily correlate well with the abnormal anatomic findings. Furthermore, these symptoms may not resolve completely following treatment. Clinicians should be mindful about this rare entity while evaluating the patients with obstructive symptoms, as timely and accurate diagnosis of SMA syndrome allows us to provide effective treatment and improve outcomes.
